# *In vitro* anti-HIV, cytotoxicity and nutritional analysis of *Trianthema portulacastrum* L. (Aizoaceae)

**DOI:** 10.1186/s12906-023-04300-5

**Published:** 2024-01-12

**Authors:** Mahboob Adekilekun Jimoh, Muhali Olaide Jimoh, Mujidat Bello, Idris Olawale Raimi, Gideon Olarewaju Okunlola, Nompumelelo Mkhwanazi, Charles Petrus Laubscher

**Affiliations:** 1 Department of Plant Biology, Osun State University, P.M.B, Osogbo, 4494 Nigeria; 2 Department of Horticultural Sciences, Faculty of Applied Sciences, Cape Peninsula University of Technology, Bellville, 7535 South Africa; 3 Department of Plant Science, Olabisi Onabanjo University, Ago-Iwoye, Nigeria; 4 National Biotechnology Development Agency, Lugbe, Abuja, Nigeria; 5 Department of Botany, Obafemi Awolowo University, Ile-Ife, Nigeria; 6 HIV Pathogenesis Programme, Doris Duke Medical Research Institute, University of KwaZulu-Natal, Durban, 4013 South Africa

**Keywords:** Aizoaceae, Antiretroviral drugs, Antiviral nutrients, Botanical ingredients, *Trianthema portulacastrum*

## Abstract

The development of antiretroviral therapy has brought a tremendous relief to the world as it minimizes mortality, reduces HIV transmission, and suppresses progression in infected patients. However, the orthodox antiretroviral therapy is faced with limitations which have necessitated a continuous search for more novel plant-based antiviral compounds, which can bypass the existing barriers created by drug resistance and target more viral proteins. Despite the edibility and enormous pharmacological benefits of *T*. *portulacastrum*, little is known about its nutrient profiles and potential use as a natural source of antiviral drug. This study focuses on the full feed analysis and anti-HIV potential of two biotypes of *T*. *portulacastrum*. Ethanolic extracts of both biotypes of *T. portulacastrum* (T01 and T02) had significant inhibitory effects on the level of replication of the HIV-1. Both extracts induced the inhibition of at least 50% of the HIV-1 viral load at considerably low IC_50_ values of 1.757 mg/mL (T01) and 1.205 mg/mL (T02) which is comparable to the AZT standard. The protein composition ranged between 8.63-22.69%; fat (1.84-4.33%); moisture (7.89-9.04%); fibre (23.84-49.98%); and carbohydrate content (38.54-70.14%). Mineral contents of tested *T. portulacastrum* varied considerably in different parts of the plant. Nitrogen N mineral ranged between 13.8-36.3 mg/g; sodium Na (2.0-14.0 mg/g); potassium K (14.0-82.0 mg/g); magnesium Mg (2.8-7.1 mg/g); calcium Ca (9.1-24.7 mg/g); phosphorus P (1.3-3.6 mg/g); iron Fe (193.5-984.0 ppm); zinc Zn (42.5-96.0 ppm); manganese Mn (28.5-167.5 ppm); and copper Cu (2.0-8.5 ppm). These mineral values are comparable or higher than values quoted for common vegetables, suggesting that *T. portulacastrum* is a nutrient-dense vegetable that could provide alternative sources of antiviral nutrients to HIV-infected individuals. Further studies are recommended to unravel key metabolites responsible for high nutrient profiles and antiretroviral effects in *T. portulacastrum*.

## Introduction

The human immunodeficiency virus (HIV) has been contracted by over 84.2 million people globally, majority of whom are resident in developing countries especially, Sub-Sahara Africa [1]. As of 2021, there were 38.4 million carriers of HIV while about 40.1 million people have reportedly died of the epidemic [1]. The burden of HIV epidemic is severely felt in the African region, accounting for about 71% of global HIV statistics [2]. Both pathogenesis and replication of the HIV RNA are determined by viral load, viral characteristics, and host response, hence, the nature of the viral genotype must be considered while developing clinical approaches to prevent or lessen the severity of acute infection [2, 3]. The detection of some microRNAs that possess binding sites within the viral genome has contributed immensely to viral pathogenesis and novel mechanisms of blocking the replication of the RNA virus and its associated vectors [4]. These microRNAs can promote genome stability, alter cell-specific miRNA levels as they promote the evasion of antiviral immune response within the host cell, leading to the development [4, 5].

Ever since HIV was first reported about 40 years ago, there has been no effective vaccine that can prevent HIV infection although significant progress has been made on clinical management of the disease [6]. The introduction of antiretroviral therapy has reduced its mortality and morbidity considerably from a deadly disease to a chronic condition [1, 3]. However, HIV-1 antiretroviral drugs are unable to inhibit the replication of the HIV-1 from the viral reservoirs [7, 8]. Therefore, new strategies are required to suppress HIV and activate the virus that hides in the latent viral reservoirs. There are potential strategies that are currently investigated to eliminate cells latently infected with HIV-1 include Shock and Kill, Lock and Block, and new approaches such as CRISPR/Cas9 gene-editing [9]. Moreover, the development of HIV-1 drug resistance also hinder the success story of antiretroviral drugs. It becomes difficult as the virus can replicate repeatedly once its DNA is incorporated into the chromatin of the host’s cell. This is further exacerbated by the establishment of functionally quiescent infection of self-renewing CD4^+^ T memory cells that coordinate immune response to infection [5, 10, 11]. The development of antiretroviral therapy has brought a tremendous relief to the world as it minimizes mortality, reduces HIV transmission, and suppresses progression in infected patients [12, 13]. The approach also serves as the mainstay of HIV management when administered effectively, as it improves life expectancy and prevents new infections that may compromise the immune system [11, 14].

Amidst the different research advances to combating the HIV menace, the antiretroviral therapy is faced with challenges such as the development of adverse effects and emergence of HIV drug resistance, incomplete elimination of HIV reservoirs in infected patients, high cost of drugs and limited access to treatment by infected rural dwellers especially, in underdeveloped countries [15, 16]. These limitations notwithstanding have only re-invigorated the continuous search for more novel plant-based antiviral compounds which can bypass the existing barriers created by drug resistance and target more viral proteins that escape the inhibitors of integrase, reverse protease, and transcriptase [17].

*Trianthema portulacastrum* (desert horse purslane, giant pigweed, black pigweed) or Olowonjeja, Akisan (Yoruba, Southwestern Nigeria) is a prostrate, diffusely branched annual plant with glabrous leaves [18–20]. It belongs to Aizoaceae, a plant family that is endemic to South Africa but widely distributed in sub-Sahara Africa, India, Southern China, tropical America, Southeast and West Asia [21, 22]. In Africa, the plant is widely found in Nigeria, Egypt, Ivory Coast, Senegal, Togo, and Gambia where it is underutilized and largely regarded as an invasive weed [21, 23, 24]. In Ayurvedic medicine, *T*. *portulacastrum* is an important herb having diverse uses in the formulation of herbal drugs used to treat cough, uteralgia, inflammation, and a valuable herb in Indian diets [18, 25] . The compounds that are found in *T. portulacastrum* Linn. is ecdysterone and the other constituents are trianthenol, 3-acetylaleuritolic acid, 5,2’-dihydroxy-7-methoxy-6,8-dimethylflavone, leptorumol, 3,4-dimethoxy cinnamic acid, 5-hydroxy-2-methoxybenzaldehyde, p-methoxybenzoic acid, and beta cyanin [26]. Different parts of *T. portulacastrum* are used traditionally as laxatives, alexiteric, analgesic, and for the treatment of breast cancer, asthma, bronchitis, cardiac diseases, hepatic complications, food poisoning, anaemia, night blindness, beriberi, corneal ulcers, dropsy, oedema, inflammation, piles, migraine, and rheumatism [25, 27, 28]. Despite its edibility [29] and enormous pharmacological benefits, little is known about its food value and its potential use as a natural source of antiviral drug. This study focuses on the full feed analysis of *T*. *portulacastrum* and its anti-HIV activity to complement existing antiretroviral remedies so that it can provide alternative sources of antiviral nutrients to infected individuals to improve their well-being and achieve a healthy generation.

## Materials and methods

### Plant collection and preparation

Fresh parts of the two biotypes of *T*. *portulacastrum* were harvested from the backyard of Olorubu Central Mosque, Olaiya Community, off Awo Road, Ede, Osun State, Southwestern Nigeria (7° 45´ 11ʺ N, 4° 25´ 34ʺ E). The plant specimen was deposited at the IFE herbarium in Department of Botany, Obafemi Awolowo University, Ile-Ife, Nigeria and allocated a voucher number IFE-18184 accordingly. After collection, the plant samples were separated into leaves, whole plant and roots for the two biotypes, resulting in six (6) different samples. The plant samples were oven-dried to a constant weight at 35°C and pulverized with an electric blender. The pulverized plant was kept in an airtight bottle that was transferred into a refrigerator for further use.

### Extraction procedure

Twenty grams of the pulverized plant was weighed in a round bottom flask containing 500 mL ethanol. The mixture was shaken vigorously at 120 rpm on an orbital shaker (Orbital Incubator Shaker, Gallenkamp) for two days, and run through a Whatman No. 1 filter paper fixed in a Buchner funnel. This suction force for the filtration process was generated by a vacuum pump connected to the Buchner funnel. Excess ethanol in the filtrate was sucked out with a rotary evaporator (Strike-202 Steroglass, Italy) kept at 78 °C until the crude extract became concentrated and dry.

### The cytotoxicity of the plant extracts

The cytotoxicity effect of the plant extracts was assessed on TZM-bl cell lines using the MTT (3-[4.5-dimethylthiazol-2-yl]-25 diphenyl tetrazolium bromide) cell proliferation assay kit according to the manufacturer's instruction (Thermo Fisher Scientific, South Africa) [17]. Briefly, 10,000 cells/well of TZM-bl cells were seeded in a 96-well plate (Costar) and incubated for 48 hours at 37°C, 5% CO_2_. Ten microliters of each ethanolic plant extract were serially diluted 10-fold from different concentrations in DMEM containing 10% heat-inactivated fetal bovine serum, 50 µg/mL Gentamycin, and 25 mM Hepes buffer in a 96-well plate. Azidothymidine (AZT) at 30 μg/mL was used as the positive control and uninfected cells as the negative control. After incubation, 10 µL MTT reagent (5 mg/mL in PBS) was added to each well and incubated for 4 hours. Afterwards, the media was replaced with a fresh DMEM medium. Then, the formazan crystals were dissolved in 50 µL 0.2% DMSO and incubated for 10 minutes. The absorbance was measured using a Victor Nivo, multimode plate reader at 540 nm (PerkinElmer Inc. USA) [30]. The results were expressed as the percentage viability of cells. The cytotoxicity concentration at 50% (CC_50_) for each extract was calculated based on the non-fit regression curve on the GraphPad Prism Software (v.5.00.288). The percentage cell viability was calculated using the formula:

$$\left(\%\right)Cell\ Viability=\frac{(Sample\ absorbance -Cell-free\ sample\ blank)}{(Mean\ media\ control\ absorbance)} \times 100$$

### Antiviral screening of plant crude extracts using luciferase-based antiviral assay

The TZM-bl cells were maintained at 37^o^ C and 5% CO_2_ in DMEM medium (containing 10% heat-inactivated fetal bovine serum, 50 µg/mL Gentamycin, and 25 mM Hepes buffer) [31, 32]. In this experiment, 10 µL each of crude extract was diluted 10-fold from 300 μg/mL in DMEM in a 96-well plate to achieve varying plant extract concentrations. In same plate, well with cells only were plated as negative cell control (CC) and well with cells and virus only were plated as virus control (VC). After that, 50 µL HIV-1 NL4.3 virus (400 TCID) was added to all wells except cell control wells and incubated fo 1 hour at 37°C, 5% CO_2_. Then, TZM-bl cell suspension prepared at a density of 10 000 cells/mL in DMEM containing Dextran (Thermo Fisher, South Africa) was seeded (10 000 cells/well) in a 96-well plate (Costar) and incubated at 37°C, 5% CO_2_ for 72 hours. Positive control was set up using a known reverse transcriptase inhibitor, azidothymidine (AZT), at a 30 μg/mL starting concentration which was also included in the experiment (Virus control). After incubation, the DMEM medium was replaced, and 100 µL BrightGlo luciferase reagent (Promega, Madison, United States) was added to each well under low light conditions and incubated at room temperature for 2 minutes to allow complete cell lysis. All the contents were transferred to a corresponding 96-well bottom flat black plate (Costar, Germany). Luminescence was read immediately in a Victor Nivo multimode microplate reader at 540nm (PerkinElmer; USA). The level of viral replication was expressed as a percentage of the HIV-1 inhibition following the equation below.

$$\%\ \text{HIV inhibition}=\frac{(\text{Average sample }-\text{Average control})}{[1-\left(\text{Average viral control}-\text{Average control}\right)]}\times 100$$

The half-maximal inhibitory concentration (IC_50_) that induced at least 50% changes in the dose-response curve was determined with a GraphPad Prism Software (v.5.00.288).

### Full feed analysis

Nutritional properties of the pulverized plant samples were analysed following referenced laboratory procedures of AOAC, (2016). The Inductively Coupled Plasma- Optical Emission Spectrometer (Varian Vista-MPX, Victoria 3170, Australia) facility in the Analytical laboratory of Department of Agriculture, Kwa-Zulu Natal was used to analyse mineral content of the tested plant.

### Proximate composition of* T. portulacastrum*

#### Ash content

The percentage of ash content in the tested plant was determined following the analytical procedure of the Association of Official Analytical Chemists (AOAC), [33]. A porcelain crucible was heated in an oven set at 105 °C for 1 hour. The bowl was cooled in a desiccator and weighed “W1”. One gram of the tested plant was measured in the crucible and reweighed “W2”. The crucible and its content were heated for 1 hour in a muffle oven set at 250 °C. To ensure that the plant sample is completely ashed, the oven temperature was adjusted to 550 °C. After 5 hours, the crucible was taken out of the furnace and cooled in a desiccator. The tested plant was weighed “W3”, and its ash content of the was determined as

1 $$\%\ \text{Ash content }=\frac{{\text{W}}2-{\text{W}}3}{{\text{W}}2-{\text{W}}1}\times 100$$

#### Crude protein

To determine the crude protein, 2 g of the tested sample was boiled in a Kjeldahl flask filled with concentrated H_2_SO_4_ (20 mL) until a clear mixture was seen [34]. The acid-digestion process was catalyzed with a digestion tablet made of 0.15 g titanium oxide, 0.15 g of copper sulfate, and 5.0 g of potassium sulfate. After digestion, the mixture was filtered, and the filtered extract was collected in a 250 mL round-bottomed flask. Fifty milliliters (50 mL) of 45% NaOH added to the filtrate, and the resulting mixture was further distilled in a 500 mL flask and 150 mL of the distilled fraction was poured in a reaction bottle already having 100 mL of 0.1 M HCl. The resulting solution was titrated with methyl orange versus 2.0 mol/L NaOH. A yellow colour change indicated the titration endpoint from which the percentage nitrogen equivalent was evaluated using the formula below.

2 $$\%\ \text{Nitrogen equivalent}=\frac{\left[\left(\text{ml std acid}\ \times\ \text{N of acid}\right)-\left(\text{ml bank}\ \times\ \text{N of base}\right)\right]-\left(\text{ml std base}\ \times\ \text{N of base}\right)\times\ 1.4007}{\text{original weight of the pulverised sample}}$$

Where N = normality, the nitrogen value is multiplied by a constant factor of ‘6.25’ to determine the percentage crude protein [35].

#### Moisture content

The moisture content was determined according to the procedure used by [36]. A blank porcelain bowl was oven-dried for one hour at 105 °C, chilled in a desiccator, and weighed ‘W1’. One gram of pulverized leaf of *T. portulacastrum* was measured in the porcelain container and reweighed ‘W2’ after oven drying at 105 °C. The bowl and its content were cooled in a desiccator and reweighed ‘W3’. The moisture content was calculated as given in the equation below.

3 $$\%\ \text{Moisture content }=\frac{{\text{W}}2-{\text{W}}3}{{\text{W}}2-{\text{W}}1}\times 100$$

#### Crude fat content

The percentage crude fat in the tested plant was determined following the analytical procedure of the Association of Official Analytical Chemists (AOAC), [33]. One gram of pulverized plant was extracted in 100 mL of diethyl ether and subjected to vigorous shaking on an orbital shaker for 24 hours. After orbital shaking, the mixture was filtered, and a pre-weighed glass beaker was used to collect the ether filtrate. Thereafter, the ether filtrate was homogenized with a measured volume of diethyl ether (100 mL), shaken vigorously for another 24 hours, filtered, and transferred in a beaker labelled ‘W1’, which was used to collect the filtrate. The ether filtrate in the beaker was vaporized and allowed to dry in a water bath and later transferred into an oven set at 55 °C and the beaker was reweighed ‘W2’. The crude fat content was calculated as given in the equation below.

4 $$\%\ \text{Crude fat content }= \frac{{\text{W}}2-{\text{W}}1}{\text{original weight of the pulverised sample}} \times 100$$

#### Neutral Detergent Fibre (NDF)

The NDF content of the pulverized *T. portulacastrum* was determined from the equation below as given by [37]

5 $$\%\ \text{Neutral Detergent Fibre }= \frac{\left({\text{W}}1+{\text{W}}2\right)-{\text{W}}1}{\text{Weight of the sample}}\times 100$$

#### The total carbohydrate content

The total carbohydrate content in the tested plant samples was determined by subtracting the sums of the total fat, crude protein, ash, and moisture from the 100% weight of the plant sample [38] using the formula below.

6 $$\%\ \text{Carbohydrate} = 100-\left(\%\ {\text{fat}}+\%\ {\text{protein}}+\%\ {\text{ash}}+\%\ {\text{moisture}}\right)$$

## Results

### Cytotoxicity of the T01 and T02 samples

The cytotoxicity analysis was performed on TZM-bl cells using MTT proliferation. The cytotoxicity of each *T. portulacastrum* (T01 and T02) crude extract was represented as the percentage of cell viability. The crude extract, T01 had a cell viability of less than 50% and TO2 was greater than 50% as the CC_50_ value was very wide T02, hence selective index not determined (Table 1). According to the United States National Cancer Institute Plant Screening Program, a crude extract is generally considered to have in vitro cytotoxic activity when the CC50 value is <30–40 μg/mL [39]. The cytotoxicity results indicate that the remaining crude extract of T01 showed no cytotoxicity on TZM-bl cells with CC_50_ value of 3.384 mg/mL while T02 was very wide and thus, could not be determined (Table 1). The positive reference, AZT, did not show cytotoxicity on TZM-bl cells at a CC_50_ value of 35.64 µg/mL. The CC_50_ is significant in these results to exclude the non-specific antiviral effect and decide the extracts' toxic concentration.

**Table 1:** Inhibitory concentrations and cell cytotoxicity of plant extracts against HIV-1 subtype B (NL4.3) in TZM-bl cell lines

**S/N**	**Plant extracts**	**Inhibitory concentration at 50% (IC**_**50**_**)**	**Cell cytotoxicity at 50% (CC**_**50**_**)**	**Selective indexes (SI) CC**_**50**_**/IC**_**50**_
1	T01	1.757 mg/mL	3.384 mg/mL	1.93
2	T02	1.205 mg/mL	Very wide	Not determined
3	AZT	0.004 µg/mL	35.64 µg/mL	8910

### The anti-HIV-1 activity of the crude extract of the *T. portulacastrum* using a luciferase-based antiviral assay

**Fig. 1:**
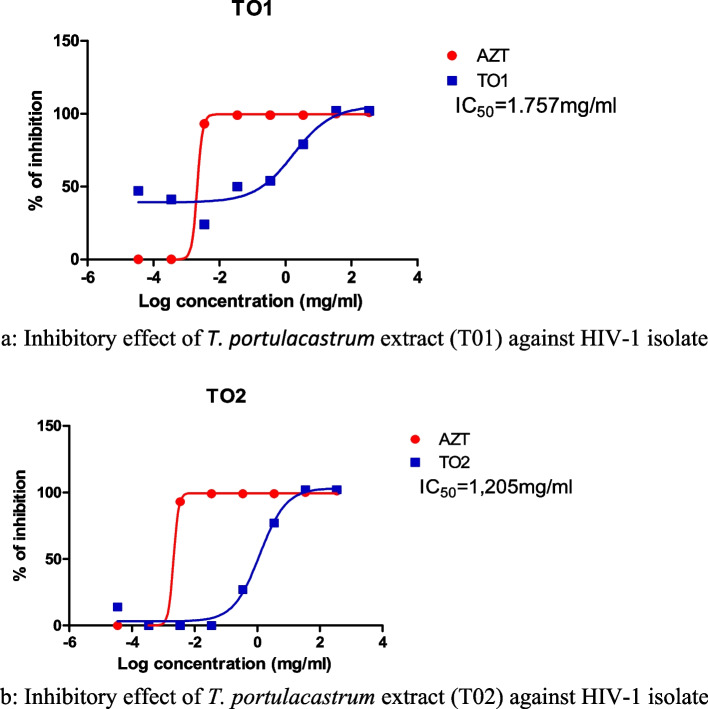
**a** Inhibitory effect of *T. portulacastrum* extract (T01) against HIV-1 isolate. **b**: Inhibitory effect of *T. portulacastrum* extract (T02) against HIV-1 isolate. **a** & **b**: Percentage inhibition curves of the *T. portulacastrum* extracts (T01 and T02) with Luciferase-based antiviral assay using TZM-bl cell lines. The TZM-bl cells were infected with HIV-1 (NL4.3) and treated with the serial dilution of *T. portulacastrum* crude extracts with AZT as positive control. The infected and treated TZM-bl cells were incubated for 48hrs at 37°C and 5% CO_2_. IC_50_ for T01=1.757 mg/mL; IC_50_ for T02=1.205mg/mL

Ethanolic extracts of both biotypes of *T. portulacastrum* (T01 and T02) respectively representing the green and purple leaved plants had significant inhibitory effects on the level of replication of the HIV-1. The IC_50_ values of 1.757 mg/mL (T01) and 1.205 mg/mL (T02) at which both extracts induced the inhibition of at least 50% of the HIV-1 viral load were considerably low and comparable to the AZT standard (Figs. 1a and b).

### Mineral analysis

The major elements determined were calcium (Ca), magnesium (Mg), potassium (K), phosphorous (P), nitrogen (N) and sodium (Na) while the minor elements examined were copper (Cu), iron (Fe), manganese (Mn) and zinc (Zn) (Table 2). There was no variability in the N content of the tested plant samples, however, variability occurred in other mineral elements. The highest Ca mineral was recorded in the whole plant of *T*. *portulacastrum* with purple leaves (T04), followed by leaves of purple flowered type (T02) while the least Ca value was obtained in the roots of purple flowered biotype although the *P*- value (0.000) obtained suggested that there is significant variability in the Ca mineral content of different parts of *T*. *portulacastrum* (T01 - T06) tested. Magnesium mineral was high in T01 and T05 respectively coding for leaves and roots of white-flowered *T*. *portulacastrum* while the least Mg value was observed in the whole plant with pink flower. However, the *P*- value (0.009) suggested that there is significant difference in the Mg content of the tested parts of *T*. *portulacastrum*. Equivalent high K content was recorded in the tested T01, T02, T03 and T05 samples while the lowest K was observed in the whole plant of white-flowered *T*. *portulacastrum*. Like Ca and Mg, there is significant variability in the K content as *P*- value of 0.000 was recorded. Compare to other minerals evaluated, no significant differences were observed in the Na of Cu content of different samples of *T*. *portulacastrum* tested. Besides, Na, Fe, Mn and Zn content varied significantly in different parts of the plant samples (Table 2).

**Table 2:** Mineral elements in *T. portulacastrum*

	**Major elements**							**Minor elements**			
**Samples**	**Ca (mg/g)**	**Mg (mg/g)**	**K (mg/g)**	**P (mg/g)**	**N (mg/g)**	**Na (mg/g)**	**K/Ca+Mg (mg/g)**	**Cu (ppm)**	**Fe (ppm)**	**Mn (ppm)**	**Zn (ppm)**
T01	12.7±0.05c	7.1±0.02a	80.3±0.04a	3.0±0.01b	36.3±0.03	14.0±0.06a	17.0±0.05a	4.0±0.00abc	448.0±15.6c	142.5±6.36ab	99.5±3.54a
T02	14.5±0.01b	6.0±0.03ab	81.5±0.12a	3.0±0.01b	33.4±0.06	11.7±0.01b	17.1±0.01a	2.5±0.71bc	639.5±26.2b	167.5±0.71a	56.0±0.00b
T03	9.1±0.07d	6.5±0.01ab	81.8±0.09a	3.6±0.01a	19.6±0.22	13.7±0.01a	21.3±0.09a	2.0±1.41c	193.5±26.2d	152.0±5.66ab	52.5±4.95b
T04	24.7±0.01a	2.8±0.18c	14.0±0.08c	1.5±0.06c	26.3±1.57	3.4±0.08c	6.4±0.06b	7.5±4.95ab	984.0±5.66a	28.5±26.20c	79.0±38.2ab
T05	12.4±0.04c	6.9±0.03a	82.0±0.28a	2.9±0.01b	19.9±0.11	13.5±0.04a	17.5±0.04a	4.0±0.00abc	436.5±6.36c	137.5±3.54b	96.0±4.24a
T06	6.0±0.01e	5.1±0.02b	27.3±0.06b	1.3±0.01c	13.8±0.06	2.0±0.01d	9.9±0.01b	8.5±0.71a	425.5±2.12c	49.5±0.71c	42.5±0.71b
**Analysis of variance**											
* P*- value	0.000	0.009	0.000	0.000	0.398ns	0.000	0.007	0.101	0.000	0.000	0.046
F- value	541.84	9.26	1048.61	27.80	1.23	329.01	10.09	3.09	507.95	53.37	4.57
Pooled StDev	0.039	0.075	0.138	0.025	1.111	0.043	0.247	2.141	16.773	11.33	15.882

### Proximate analysis

There were no significant differences in the protein, fat and moisture content of the tested plant materials. However, the ash, acid detergent fibre (ADF) and NDF contents varied considerably (Table 3). The highest values of ADF and NDF were recorded in roots of pink-flowered *T*. *portulacastrum* (T06) although an equivalent ADF value was recorded in the T05 and T06 root samples of *T*. *portulacastrum*.

**Table 3:** Proximate content of different parts of *T. portulacastrum*

**Samples**	**ADF (mg/g)**	**Ash (mg/g)**	**Moisture %**	**NDF (mg/g)**	**Fat (mg/g)**	**Protein (mg/g)**	**Carbohydrate (mg/g)**
T01	238.4±1.14c	265.8±0.64a	8.39±0.15	549.1±1.32c	38.0±0.09	226.9±0.18	385.4±0.27d
T02	249.0±0.06c	260.9±0.63a	7.86±0.27	525.8±0.59cd	18.4±2.06	208.6±0.37	433.5±0.83c
T03	245.0±0.52c	240.2±0.14ab	8.48±0.07	501.4±0.25d	29.8±0.15	122.6±1.39	522.6±0.44bc
T04	307.6±5.18b	179.0±7.50bc	8.57±1.39	499.8±3.68d	43.3±2.34	164.8±0.98	527.2±3.05bc
T05	449.5±0.64a	115.8±0.21cd	9.04±0.04	792.4±1.41b	19.2±0.14	124.9±0.71	649.7±0.28b
T06	499.8±0.74a	101.8±0.20d	8.34±0.13	847.0±0.74a	27.1±0.04	86.3±0.35	701.4±0.18a
**Analysis of variance**							
*P*- value	0.000	0.005	0.555ns	0.000	0.403ns	0.398ns	0.004
F- value	53.86	11.26	0.86	162.92	1.22	1.23	12.35
Pooled StDev	2.210	3.074	0.584	1.742	1.278	6.944	4.035

## Discussion

The invention of antiretroviral therapy has made HIV manageable, and this has changed its status from a deadly disease to a controllable chronic disease [13, 40]. However, the challenges of drug resistance [41, 42], high cost of drugs, restricted access to orthodox treatment, partial elimination of viral load and other limitations associated with antiretroviral therapy have necessitated the invention of alternative therapy from plant-based remedies for the treatment of chronic diseases such as HIV-1 [13]. It has been reiterated in literature that plants are depots of biologically active chemicals, and plant-based drugs could offer safer, affordable, and sustainable means of treating various degenerating ailments [43–45].

In addition, the development of food supplements from plants could enhance bioavailability of essential nutrients needed to boost the body immune system and combat nutrient deficiencies [46–48]. In effect, this may create the much-needed synergy between different baseline minerals present in plants [49] and useful bioactive compounds such as epigallocatechin, capsaicinoids, lycopene, terpenoids, phenolics, flavonoids, carotenoids, quercetin required to improve the nutraceutical and functional values of foods [50–53]. These botanical ingredients are useful as natural drugs, functional foods, dietary supplements, food fortificants, and dietary markers for healthy living [54–56] and are abundant in large quantities in *Papaver somniferum* L., *Terminalia chebula* Retz.*, Asparagus racemosus* Willd., and *Terminalia* *hadleyana* W. Fitzg. among other plants that are renown for anti-HIV potency [57–59].

Bioavailability of antiviral botanical ingredients in *T. portulacastrum* as revealed by this study has provided a plausible answer to the accelerated search for novel antiretroviral botanicals [60, 61], an indication that the species could be useful for the formulation of antiviral foods. Among the principal chemical constituents of *T. portulacastrum* are ecdysterone, trianthenol, 3-acetylaleuritolic acid, 5,2'-dihydroxy-7-methoxy-6,8-dimethylflavone, leptorumol, 3,4-dimethoxy cinnamic acid, 5-hydroxy-2-methoxybenzaldehyde, p-methoxybenzoic acid, and beta cyanin [18, 26]. It is not known which one possesses the antiviral activity, also the mineral compositions and its medicinal properties is yet to be investigated. This approach will boost life expectancy in HIV-infected patients, promote their survival [61] and complement existing therapies recommended for HIV management if fully integrated [54, 62]. Since ethanolic extracts of both biotypes of *T. portulacastrum* inhibited the replication of the HIV-1 by 50% at IC_50_ values of 1.757 mg/mL (T01) and 1.205 mg/mL (T02), it is evident that *T. portulacastrum* has antiretroviral precursors, and may be explored further for more synergistic antiretroviral therapeutic options. The mechanism of HIV-1 inhibitory properties of *T. portulacastrum* is not established, hence, further studies may be required to investigate these findings.

The proximate and mineral composition of *T. portulacastrum* was reported by Khan et al. [29] However, nutritional assessment of different biotypes of the species remains unknown. In this study, the densities of reference minerals and proximate content of two biotypes of *T. portulacastrum* harvested from Southwestern Nigeria were profiled. Results showed that nutrient densities varied in different parts of *T. portulacastrum* and might be used as a natural source of antiretroviral nutrients. Also, compared to some known vegetables, *T. portulacastrum* has a rich proximate and nutrient profiles [63]. This is evident in previous findings on nutrient profiles of *Spinacia oleracea* L. [64], *Amaranthus caudatus* L. [65], *Celosia argentea* Linn. [37] *Solanum nigrum* L. [66], *Brassica oleracea* L. [63] *Trachyandra divaricata* (Jacq.) Kunth [34, 36], grain amaranth [67], *Rumex crispus* L. [68], *Siphonochilus aethiopicus* (Schweinf.) B.L.Burtt [69], among others.

Compared to other plants such as *Papaver somniferum* L., *Terminalia chebula* Retz.*, Asparagus racemosus* Willd., and *Terminalia* *hadleyana* W. Fitzg. renown for anti-HIV potency [57–59], the proximate content and nutritional profiles of *T. portulacastrum* varied considerably. Kohli et al. [70] earlier reported 6.1 % protein; 6.2% fat; 52.89% carbohydrate; 9.5% moisture, and 17.3% fibre in *A. racemosus* which are relatively lower than values obtained in the analyzed samples of *T. portulacastrum* with higher protein composition ranging between 8.63-22.69%; lower fat (1.84-4.33%); equivalent moisture (7.89-9.04%); higher fibre (23.84-49.98%); and comparable carbohydrate content (38.54-70.14%). Mineral contents of tested *T. portulacastrum* were higher than antiviral fruit of *Terminalia* *hadleyana* as reported by Zhang et al. [71], suggesting that *T. portulacastrum* is an underestimated functional food given its elevated nutrient profiles. Findings from this study further suggest that *T. portulacastrum* may be explored further to unravel its potent antiviral precursors.

## Conclusion

*Trianthema portulacastrum* is an underutilized edible vegetable with elevated nutrient profiles. Findings from this study revealed the anti-HIV potential of *T. portulacastrum* which may be attributed to high concentrations of essential trace elements such as Fe and Zn in the plant. This potential may be exploited in the development of antiretrovirals while complementing existing HIV-management therapies. Further studies are recommended to unravel key metabolites responsible for antiretroviral effects in *T. portulacastrum*.

## Data Availability

All data is included in the manuscript. All reasonable requests for materials used should be directed to the corresponding author.
